# First Detection of Cryptosporidium Canis and Occurrence of *Cryptosporidium* spp. in Hospitalized Patients in Romania

**DOI:** 10.3390/microorganisms13040931

**Published:** 2025-04-17

**Authors:** Rodica Georgiana Dărăbuș, Mirela Imre, Gheorghe Dărăbuș, Marius Stelian Ilie, Alexander Tudor Olariu, Diana Maria Dărăbuș, Voichița Lăzureanu, Ovidiu Roșca, Tudor Rareș Olariu

**Affiliations:** 1 Discipline of Parasitology, Department of Infectious Diseases, Victor Babes University of Medicine and Pharmacy, 300041 Timisoara, Romania; georgiana.darabus@umft.ro (R.G.D.); rolariu@umft.ro (T.R.O.); 2 Center for Diagnosis and Study of Parasitic Diseases, Department of Infectious Disease, Victor Babes University of Medicine and Pharmacy, 300041 Timisoara, Romania; 3 Discipline of Parasitology, Faculty of Veterinary Medicine, University of Life Sciences “King Mihai I” from Timisoara, 300645 Timisoara, Romania; mirela.imre@usvt.ro (M.I.); mariusilie@usvt.ro (M.S.I.); 4 Patogen Preventia, 300124 Timisoara, Romania; alexanderolariu@yahoo.com; 5 Oftalmo Sensory-Tumor Research Center—ORL (EYE-ENT), Ophtalmology Department “Victor Babeș” University of Medicine and Pharmacy, 300041 Timisoara, Romania; 6 Infectious Diseases Clinic, Victor Babes University of Medicine and Pharmacy, 300041 Timisoara, Romania; lazureanu.voichita@umft.ro; 7 Methodological and Infectious Diseases Research Center, Department of Infectious Diseases, Victor Babes University of Medicine and Pharmacy, 300041 Timisoara, Romania; ovidiu.rosca@umft.ro

**Keywords:** *C. parvum*, *C. canis*, Romania, occurrence, risk factors, sequencing

## Abstract

The study aimed to identify *Cryptosporidium* infection in hospitalized patients in Western Romania. Stool samples were collected from 175 patients, both male and female, aged between 2 months and 88 years, residing in urban and rural areas. The identification of *Cryptosporidium* was performed using a qualitative chromatographic rapid test, supplemented by the modified Ziehl–Neelsen method described by Henricksen and Pohlenz. *Cryptosporidium* spp. were identified through PCR analysis and Sanger sequencing. To assess potential risk factors for cryptosporidiosis, a questionnaire was administered to the study participants. Laboratory test results revealed a cryptosporidiosis occurrence of 7.42%, with a significantly higher occurrence observed in urban areas (*p* < 0.05). Two species were identified, *C. parvum* and *C. canis*, the latter being reported for the first time in humans in Romania. Among the assessed risk factors, only the area of residence significantly influenced the occurrence of *Cryptosporidium* infection. The other evaluated risk factors—age, sex, concomitant microbial infections, contact with animals, use of public transport, international travel, frequenting children’s playgrounds, and swimming pools—although potentially involved in *Cryptosporidium* infection, did not have a significant contribution. This study represents the first report of *C. canis* identified in humans in Romania. Our results indicate a high occurrence of human cryptosporidiosis in hospitalized patients, with a significantly higher rate observed in individuals residing in urban areas.

## 1. Introduction

*Cryptosporidium* is an intracellular parasitic protozoan, taxonomically classified within the Order Eucoccidiorida, Phylum Apicomplexa. The parasite was first identified in 1907 in the gastric glands of mice by Tyzzer [1], and it was named *Cryptosporidium muris*. Subsequently, the parasite has been identified in various species of mammals, birds, fish, and reptiles.

Nime et al. (1976) identified *Cryptosporidium* for the first time in humans with enterocolitis. Initially, only one species was recognized—*Cryptosporidium parvum* [2]. Currently, at least 44 species and over 120 genotypes are recognized [3]. Among these, nearly 20 species of *Cryptosporidium* are capable of infecting humans, with *Cryptosporidium hominis* and *C. parvum* being the most commonly detected species [3,4].

Recent studies have reinforced the significant role of *Cryptosporidium* as a leading cause of childhood diarrhea globally. For instance, a study published in BMC Infectious Diseases reported that *Cryptosporidium* infections are highly prevalent in children under five with moderate to severe diarrhea in Blantyre, Malawi. Additionally, the Global Enteric Multicenter Study (GEMS) identified *Cryptosporidium* spp. as the second leading pathogen associated with moderate-to-severe diarrhea in children under two years old and the leading pathogen associated with death in toddlers aged 12 to 23 months. These findings underscore the critical impact of *Cryptosporidium* on child health, highlighting the need for targeted interventions and preventive measures [5,6].

According to the European Centre for Disease Prevention and Control (ECDC), in Europe, children aged 0 to 4 years have the highest infection rate, with 6.4 cases per 100,000 inhabitants [7]. However, as highlighted in ECDC reports, obtaining a clear epidemiological overview of cryptosporidiosis in Europe is challenging, as many countries do not regularly issue reports on this disease. Moreover, *Cryptosporidium* is a major cause of waterborne outbreaks, alongside *Giardia*, and ranks fifth among 24 foodborne parasites in terms of significance [8,9].

Several studies indicate a growing research interest in determining the zoonotic potential of various *Cryptosporidium* spp. isolated from domestic and wild animals. These studies aim to identify the species involved in human infections, as well as the sources of infection and associated risk factors [10,11]. Recent studies [12,13,14,15,16,17] have highlighted the importance of risk factors associated with *Cryptosporidium* infection in humans, with variations depending on the level of development of the countries where cryptosporidiosis has been identified.

In Romania, several *Cryptosporidium* spp. have been identified in animals, including *C. parvum*, *C. ubiquitum*, and *C. xiaoi* [18], as well as in humans, such as *C. parvum* and *C. ubiquitum* [19]. Furthermore, *C. canis*, a known zoonotic species, was genetically characterized and identified in rivers in Western Romania by Imre et al. [20,21].

The aim of this study was to evaluate the occurrence of cryptosporidiosis in hospitalized patients in Romania and to identify the zoonotic species of *Cryptosporidium*.

## 2. Materials and Methods

Since the purpose of this research was to determine the prevalence of cryptosporidiosis, *Cryptosporidium* spp., and certain risk factors, a cross-sectional observational epidemiological study was conducted.

*a.*  *Samples and Data Collection*

One hundred seventy-five fresh stool samples were collected in stool sample containers and transported to the laboratory for examination between 7 February and 9 April 2024. The samples were collected from all individuals hospitalized during that period at the Victor Babeș Infectious Diseases Hospital in Timișoara, Western Romania. Sample collection was carried out in the first days after hospitalization. The stool samples were delivered to the laboratory in specially purchased containers for stool collection. The collection was carried out individually by each patient and, for children, by authorized hospital personnel. Coprological examinations were performed on the day of collection.

*b.*  *Study population*

The following exclusion criteria were taken into account: patient non-acceptance and if the samples were unsuitable. Because all patients agreed to the collection of samples, all patients hospitalized during the same period were included in the study. The patients, both females and males, ranged in age from two months to 88 years and were divided into seven age categories: <1 year; 1–5 years; 6–10 years; 11–17 years; 18–39 years; 40–59 years; and ≥60 years. The hospitalized patients were residents of urban and rural areas in Western Romania.

At the time of sample collection, the patients either exhibited or did not exhibit digestive symptoms. The patients were diagnosed with various pathologies that could potentially represent risk factors for *Cryptosporidium* infection. The patients included in the study received treatment for the primary condition for which they were hospitalized.

*c.*  *Cryptosporidium diagnosis tests*

The diagnosis of human cryptosporidiosis was conducted using rapid tests and confirmed by the Ziehl–Neelsen staining method modified by Henriksen. Subsequently, the positive samples were analyzed by PCR to identify the *Cryptosporidium* spp. circulating in the human population of Western Romania.

### 2.1. CerTest Crypto

CerTest Crypto is a qualitative chromatographic rapid test designed for the identification of *Cryptosporidium* oocysts in stool samples. The test demonstrates a sensitivity and specificity of over 99% (CerTest BIOTEC S.L, San Mateo de Gállego, Zaragoza, Spain) [22]. The test was performed following the manufacturer’s instructions.

### 2.2. Ziehl–Neelsen Staining Modified by Henriksen and Pohlenz

The Ziehl–Neelsen staining method, modified by Henriksen and Pohlenz was applied to all 175 fresh stool samples submitted to the CerTest Crypto analysis. This method is considered the reference method for *Cryptosporidium* diagnosis [23].

The samples were examined under an X100 optical microscope with oil immersion. *Cryptosporidium* oocysts appeared as round or ovoid elements with a red coloration against a bluish-green background, with a size of 4–5 µm. Cells, bacteria, and yeasts stained green and were easily distinguishable from *Cryptosporidium* oocysts.

### 2.3. Molecular Assay

PCR was performed from rapid test/ microscopy positive stool samples, kept at 4 °C in a mixture of 2.5% potassium dichromate solution. The first four positive samples identified by the rapid test/ method, Ziehl–Neelsen microscopy, were subjected to genetic determinations. The reactions were performed according to the technique described by Xiao et al., 1999, 2001 and Fayer and Xiao, 2008 [24,25,26] with minor modifications requested by the PCR mixture used in the reactions.

The extraction and purification of DNA from the stool sample for the detection of pathogens was carried out using the E.Z.N.A^®^ Stool DNA Kit (Omega Bio-tek, Norcross, GA, USA). In order to identify the *Cryptosporidium* spp., the amplification was performed by “nested PCR”, in two stages: primary PCR and secondary PCR [24,25,26].

Primary PCR was based on amplification of the SSU-rRNA gene of ~1325 bp size. The primers used were SSU-F2-5′-TTCTAGAGCTAATACATGCG-3′ and SSU-R2-5′CCCATTTCCTTCG-AA ACAGGA-3′. A Master Mix MyTaqTM Red Mix (BIOLINE Meridian Bioscience Inc. UK Ltd., London, UK) was used to perform the reaction.

In the secondary PCR stage, the amplified fragment size was estimated to be ~850 bp, and the primers used were SSU-F3-5′-GGAAGGGTGTATTTATTAGATAA AG-3′ and SSU-R4-5′-CTCATAAGGTGCTGAAGGAGTA-3′ [24,25,26].

In order to evaluate the results, the amplicons were visualized for the correct length using electrophoresis on a 1.5% TBE-based gel in the presence of RedSafe™ Nucleic Acid Staining Solution (iNtRON^®^ Biotechnology Inc., Seongnam-si, Gyeonggi-do, Republic of Korea) at a voltage of 120 V and 90 mA, for 60 min, using the molecular standard of 100-bp (BIOLINE Meridian Bioscience Inc.^®^ UK Ltd., London, UK). The size of the amplified fragment was ~850 bp in the case of the genus *Cryptosporidium.*

The samples were cleaned using the commercial kit ISOLATE II PCR and Gel Kit (Bioline, Meridian Bioscience Inc., London, UK) according to the manufacturer’s protocol. To determine the species, both PCR products were sequenced (Sanger sequencing) in the forward and reverse direction by the company Macrogen^®^ Europe B.V., Amsterdam, The Netherlands. Homology search was performed using the online version of the Basic Local Alignment Search Tool (BLAST) and compared with those available in the GenBank database, (https://blast.ncbi.nlm.nih.gov/Blast.cgi, accessed on 2 February 2025). Sequences with >98% similarity to a known species were considered the same taxon.

Sequencing showed that the pathogen identified in stool samples was similar to the sequences of *C. parvum* and *C. canis* and has been deposited in GenBank under accession numbers PQ047137 and PP979614 (For *C. parvum*) and PP979613.1 and PP979610.1 (For *C. canis*) (Supplementary Material S1). Phylogenetic analyses were performed using Phylogeny.fr “one-click analysis” (https://www.phylogeny.fr/, accesed on 24 March 2025) Sequences were aligned with ClustalW (v2.1). After alignment, ambiguous regions (i.e., containing gaps and/or poorly aligned) were removed with Gblocks (v0.91b). The phylogenetic tree was reconstructed using the maximum likelihood method implemented in the PhyML program (v 3.1/3.0 aLRT, https://www.phylogeny.fr/, accesed on 24 March 2025) [27]. The default substitution model was selected assuming an estimated proportion of invariant sites (of 0.000) and 4 gamma-distributed rate categories to account for rate heterogeneity across sites. The gamma shape parameter was estimated directly from the data (gamma = 2.391). Reliability for internal branch was assessed using the aLRT test (SH-Like). Graphical representation and edition of the phylogenetic tree were performed with TreeDyn (v198.3) [27].

*d.*  *Risk factors analysis*

To analyze the potential risk factors, study participants—including patients or the parents/guardians of children—completed a comprehensive questionnaire. The questionnaire gathered information on key variables, including the area of residence, sex, reported digestive symptoms, contact with animals, handwashing practices after contact with animals, and whether the water consumed was potable. For the purpose of this study, contact with animals was defined as either ownership of animals or incidental interactions with various animal species not owned by the patients.

The questionnaire was designed with input from a panel of specialists, including epidemiologists, medical doctors, veterinarians, and infectious disease experts. Additionally, its development was guided by established guidelines and documents from the European Centre for Disease Prevention and Control [7] on cryptosporidiosis. This approach ensured that the questionnaire was both relevant and aligned with international standards for assessing risk factors associated with the disease.

*e.*  *Statistical Analysis*

Statistical analyses were conducted using EPI Info v.7.2.6 (CDC, Atlanta, GA, USA, 2023). To compare differences between *Cryptosporidium*-positive and *Cryptosporidium*-negative groups, Mantel–Haenszel and the two-tailed Fisher exact tests were used. A *p*-value of less than or equal to 0.05 was considered statistically significant [28].

Statistical Analysis and Multivariate Results. To control for potential confounding variables, a multivariate logistic regression model was applied. Variables included in the model were those with a *p*-value < 0.20 in the univariate analysis or deemed epidemiologically relevant (e.g., area of residence, sex). Adjusted odds ratios (aOR), 95% CIs, and *p*-values were calculated using the Wald test. A *p*-value < 0.05 was considered statistically significant. Statistical analyses were performed using Python (statsmodels v0.14.0) and additionally IBM SPSS Statistics, version 20.0.

*f.*  *Ethical approval*

The study received prior approval from the Ethics Committee of Victor Babeș University of Medicine and Pharmacy in Timisoara (Approval Number: 12/17.03.2023). Written informed consent was obtained from all participants or, in the case of minors, from their parents or legal guardians before their enrollment in the study.

## 3. Results

*Cryptosporidium* was identified in 13 (7.42%) out of the total 175 examined patients using the CerTest Crypto. The same results were obtained using the Ziehl–Neelsen method (Table 1).

The protozoan was detected across all age groups, except for the 11–17-year age group. This finding is considered inconclusive due to the small number of cases studied (Table 2). The highest occurrence was observed in the 40–59-year age group. However, no statistically significant differences in occurrence were noted between the age groups.

An assessment of the occurrence and potential risk factors for cryptosporidiosis is presented in Table 3. *Cryptosporidium* was found to be significantly more prevalent among participants residing in urban areas (14.28%) compared to those in rural areas (4.8%) (*p* < 0.05). No significant differences in occurrence were observed between the sex of patients.

Of the 175 patients investigated, 94 had diarrhea, and 7 of these were infected with *Cryptosporidium*. Notably, *Cryptosporidium* was also identified in patients who did not report having diarrhea.

Contact with dogs and other animals was identified as a not statistically relevant risk factor for cryptosporidiosis, with an occurrence of 8.97% among patients who reported such contact (four from rural and three from urban areas). Handwashing habits were assessed by questionnaire, with most patients reporting regular handwashing after contact with animals. Despite this, 7.41% of those who reported washing their hands tested positive for the infection. A patient who admitted not washing their hands after contact with animals also tested positive.

Most of the patients stated that they use a potable water source. However, 9.82% have tested positive for *Cryptosporidium*. There were also patients who either did not know if the water they consumed was checked for potability or stated that the water was not checked.

The multivariate logistic regression model did not identify any statistically significant associations with testing positive for *Cryptosporidium.* Although not statistically significant, individuals who did not know or reported not drinking tested potable water showed a trend toward a lower risk (adjusted OR = 0.29, 95% CI: 0.06–1.39, *p* = 0.121). Female sex, digestive symptoms, and handwashing behavior were also not significantly associated with positivity. The full results are shown in Table 4.

**Figure 1:**
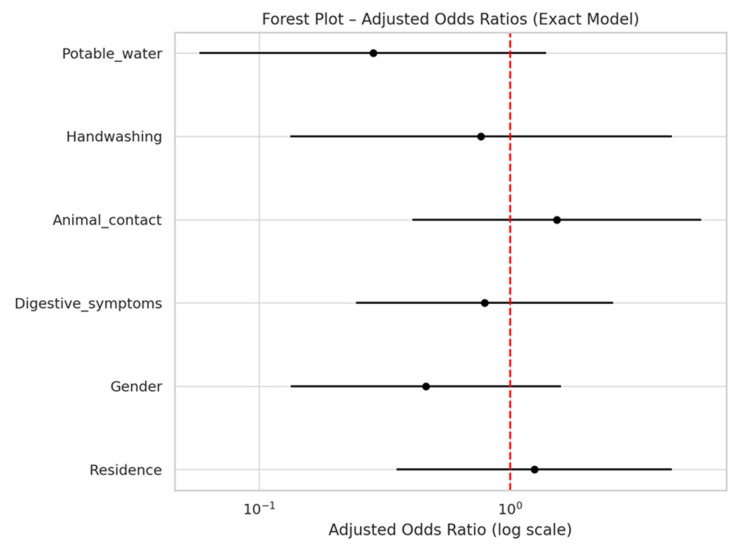
Forest plot for risk factors associated with testing positive samples. The vertical red dashed line in the forest plot represents the null value for the odds ratio (OR = 1.0). An OR of 1 indicates no association between the variable and the outcome. Confidence intervals (CIs) that cross this line suggest that the effect is not statistically significant at the 0.05 level.

Figure 1 shows a forest plot illustrating the adjusted odds ratios (ORs) and 95% confidence intervals (CIs) for risk factors associated with testing positive. The vertical red dashed line represents the null value (OR = 1.0).

Table 5 presents the pathologies for which the study participants were admitted to the hospital. The patients had a range of conditions, including enterocolitis of varying etiologies, febrile syndromes, and HIV.

Among the patients with diarrhea who tested positive for two *Cryptosporidium* species, four were men and two were women. Five of these patients were from rural areas, while only one was from an urban area. The age of positive patients ranged from 10 months to 78 years.

Table 6 summarizes the epidemiological characteristics of patients in whom two *Cryptosporidium* spp. were identified: *C. parvum* and *C. canis*. These species were detected in both rural and urban areas.

Among the four cases, three reported direct contact with animals. In contrast, in Case 4, there were no reports regarding any direct contact with animals. However, responses to the questionnaire in this case revealed that the patient was frequently engaged in activities such as visiting children’s playgrounds and swimming pools, using public transportation, and traveling internationally—activities that may represent potential risk factors for cryptosporidiosis.

All four patients were diagnosed with other infectious diseases as their primary condition, and only half presented with diarrhea. Table 5 also indicates that all patients adhered to basic hygiene practices concerning food and drinking water. Notably, none of the patients had prior knowledge about cryptosporidiosis.

**Figure 2:**
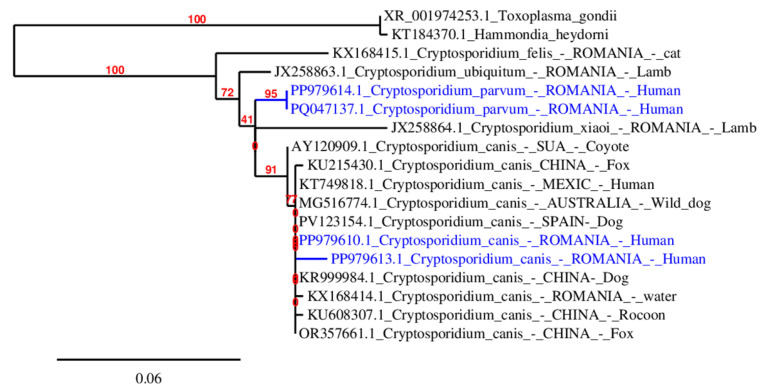
Phylogenetic relationships between *Cryptosporidium* spp. detected in humans in this study (highlighted in blue) and other *Cryptosporidium* available in GenBank using an ML analysis of partial sequences of 18S rRNA. *Toxoplasma gondii* (XR001974253) and *Hammondia heydorni* (KT184370) were set as out-groups.

*Cryptosporidium canis* isolates from different geographic locations analyzed in the generated phylogenetic tree (Figure 2) (China, Romania, Spain, Australia, Mexico, and the USA) cluster together, showing minor genetic variations based on host species (fox, dog, human, raccoon, etc.). A closely related clade contains *Cryptosporidium parvum* from Romanian human samples, suggesting a divergence between these species Outside the *Cryptosporidium* group, *Hammondia heydorni* and *Toxoplasma gondii* serve as outgroups.

## 4. Discussion

Cryptosporidiosis remains an under-explored disease in humans in Romania. This study aimed to investigate the occurrence of cryptosporidiosis and analyze the potential risk factors associated with infection. The 7.42% occurrence may be attributed to the fact that the patients included in the study had various other pathologies, potentially increasing their susceptibility to *Cryptosporidium* infection.

Similarly, a study conducted in Ethiopia reported a cryptosporidiosis prevalence of 11.5% among hospitalized patients, based on the examination of 122 stool specimens. Human samples were obtained through systematic random sampling, and fecal samples were analyzed using a modified Ziehl–Neelsen staining technique [29]. Globally, the prevalence of cryptosporidiosis is estimated at approximately 7.6%, with rates of 4.3% in developed countries and 10.4% in developing countries. Notably, much higher prevalence rates, such as 69.6%, have also been reported in specific populations or settings. This high prevalence refers to individuals from low-income countries, those with gastrointestinal symptoms, individuals under the age of five, and residents living outside urban areas. [30].

A comprehensive study on the prevalence of cryptosporidiosis in 10 Eastern European countries, conducted over a variable period of time depending on the country (1–22 years), revealed variable results. Thus, the prevalence ranged from 0% in Croatia and Bosnia and Herzegovina to 0.03% in Hungary, 1.53% in Slovenia, 3.34% in Estonia, and 4.04% in Romania. In the Czech Republic, the prevalence was 10.71% among AIDS patients. Additionally, in Serbia, cryptosporidiosis was detected (by the indirect ELISA technique) in 10.5% of HIV patients with gastrointestinal issues. In Poland, a prevalence of 14.63% was identified among patients with diarrhea (by microscopic analysis of fecal smears via modified Ziehl–Neelsen staining and IFT), all of whom were under 4 years old [31]. The comparison of the prevalence identified in our study is challenging due to the highly variable diagnostic methods and the significantly different epidemiological context of the human cohort under investigation. Compared to the situation in Eastern Europe, we identified a higher prevalence than that reported in Croatia, Bosnia and Herzegovina, Hungary, Slovenia, Estonia, and even other reports from Romania. However, our findings indicate lower prevalence rates than those reported in Serbia and Poland though under different epidemiological conditions.

The occurrence reported in this study is higher than those typically reported for Europe. This discrepancy could be attributed to pre-existing pathologies in the studied patients and to the differences in testing/reporting methods across Europe.

The fluctuation of cryptosporidiosis occurrence based on age is challenging to be explained in relation to this potential risk factor. Age may act as a risk factor, as this study found higher infection rates in children aged under 10 years and in individuals over 40 years, though these differences lacked statistical significance. The reduced occurrence in infants (<1 year) could be due to reduced exposure/transmission opportunities.

A review on cryptosporidiosis indicates that in developed countries, cryptosporidiosis occurs across all ages and regardless of immune status. In contrast, in underdeveloped countries, the infection is more prevalent in children and HIV-positive individuals. Of course, these reports regarding the relationship between HIV status and age should be interpreted with some caution. Among children, cryptosporidial infections are typically identified after the age of 2 years in developed countries, while in medium and underdeveloped countries, infections are more common in children under 2 years [16].

The occurrence of cryptosporidiosis in this study was significantly higher among urban patients, likely due to more diverse sources of infection. Similarly, previous research has reported significantly higher infection rates in urban areas (23%) compared to rural areas (12.5%) [15]. These findings have been attributed to factors such as higher population density, inadequate sanitation, and certain pathologies associated with cryptosporidiosis [13,15,32,33].

Although the occurrence of cryptosporidiosis was higher among patients who had contact with animals, the differences were not statistically significant. Previous studies have shown that sources related to agricultural activities, such as domestic animal feces and manure, may pose significant risk factors for cryptosporidiosis [12,15].

In the present study, the existence of risk factors was initially assumed, as all patients were hospitalized for various infectious diseases (Table 3). These infectious diseases could act as risk factors contributing to the progression of cryptosporidiosis. Under these conditions, additional secondary factors were analyzed, including area of residence, age, sex, presence of diarrhea, and contact with animals.

The overall occurrence of *Cryptosporidium* infection was 7.42%, varying between patients with diarrhea 7/94 (7.5%) and those without diarrhea 6/81 (7.4%). The similar occurrence rates between patients with and without diarrhea are difficult to explain. However, it is known that *Cryptosporidium* infection does not always manifest with diarrhea [14,34,35]

A study conducted on 1938 children in Gambia (using commercial immunoassay-Tech Lab, Inc., Blacksburg, VA, USA) identified a higher prevalence of *Cryptosporidium hominis* infection in those with diarrhea (12.0%) compared to those without diarrhea (4.8%). The presence of the parasites was associated with risk factors such as the consumption of stored drinking water and contact with specific animal species, including cows, cats, and rodents [14].

An extensive study on the evolution of cryptosporidiosis, based on the level of development of countries, revealed differing risk factors [16]. In developing countries, these included international travel, contact with people and animals, and swimming. In less developed countries, risk factors were linked to poor hygiene, overcrowding, and cases of diarrhea within the household. In addition, a study conducted in China highlighted the significance of Human Immunodeficiency Virus (HIV) infection as a major risk factor for cryptosporidiosis [12].

In contrast to the findings of previous studies, our research (Table 4) identified distinct potential risk factors associated with the four cases diagnosed with two *Cryptosporidium* spp. These factors included microbial diseases, contact with animals, use of public transportation, and international travel. Less commonly, activities such as visiting swimming pools and children’s playgrounds were noted as potential risks.

Interestingly, food and personal hygiene practices did not seem to significantly contribute to disease transmission, as basic hygiene norms were reportedly observed. However, a lack of awareness and knowledge about cryptosporidiosis may play a role in increasing the risk of infection.

This study marks the first identification of *Cryptosporidium canis* in humans in Romania, highlighting its potential zoonotic role. The presence of *C. canis* in humans indicates that pets can serve as a source of infection. This is demonstrated by the fact that the positive patients in the present study had contact with dogs. The fact that this species has not yet been identified in Eastern Europe does not mean that it does not exist. However, the species has been reported in Sweden [36], China [37], Mexico [38], Peru, the USA, and Kenya [39]. It is well-established that species such as *C. hominis*, *C. parvum*, *C. meleagridis*, *C. canis*, and *C. felis* are commonly found in humans [3].

Phylogenetic tree analysis of our isolates illustrates the evolutionary relationships among various *Cryptosporidium* species and related protozoan parasites confirming their more distant evolutionary relationship. Bootstrap values indicate varying confidence in the branching structure, with the highest support for deeper evolutionary separations. The *Cryptosporidium canis* isolates from humans in Romania (PP979613.1, PP979610.1) cluster closely together and are more related to *C. canis* from dogs in Spain and wild dogs in Australia, indicating potential zoonotic transmission. *Cryptosporidium parvum* isolates from humans in Romania (PQ047137.1, PP979614.1) form a distinct and well-supported clade, separate from *C. canis*, reinforcing their species-level differentiation. These findings highlight the zoonotic potential and genetic diversity of *Cryptosporidium* species infecting humans.

The development of molecular epidemiology studies, particularly in countries with a high potential for transmission, is essential for better understanding this important zoonotic pathogen [3].

The observed significant difference in the occurrence of cryptosporidiosis between urban and rural areas highlights the necessity of investigating environmental and behavioral factors contributing to this pattern. Understanding these disparities can provide crucial insights into disease transmission dynamics, allowing for the development of targeted public health interventions. To mitigate the risk of cryptosporidiosis, preventive strategies should be tailored to address the specific environmental and behavioral factors associated with its transmission. Future research should focus on assessing the effectiveness of these preventive measures in various settings to further refine control strategies.

This study has several limitations that should be acknowledged. First, the number of patients included was relatively small, which may limit the statistical power and generalizability of the findings. Second, the study was conducted over a short period of time, potentially limiting the ability to capture seasonal variations or longer-term trends in infection rates or pathogen characteristics. Third, the study population consisted exclusively of hospitalized patients, which may introduce a selection bias. As a result, the findings may not accurately reflect the prevalence or characteristics of Cryptosporidium infections in the general population, particularly among individuals with milder or asymptomatic infections who do not seek hospital care.

These limitations underscore the need for future research involving larger, more diverse populations over extended periods of time. Community-based surveillance and studies that include both symptomatic and asymptomatic individuals could provide a more comprehensive understanding of Cryptosporidium epidemiology. Additionally, expanding the scope beyond hospitalized patients would help inform more effective public health strategies for prevention, early detection, and control of the disease across different population groups.

## 5. Conclusions

In Western Romania, *Cryptosporidium* was identified in 7.42% of hospitalized patients. Two species were detected: *C. parvum* and *C. canis*, the latter being reported in humans for the first time in Romania. Genetic analysis emphasizes the zoonotic potential and genetic diversity of *Cryptosporidium* species infecting humans. Significant differences in prevalence were observed between patients from urban and rural areas. Further investigations and additional studies, expanded to other population groups, are necessary to better understand the significance of the identified risk factors and the role of infection with different *Cryptosporidium* spp.

## Figures and Tables

**Table 1:** Testing/analysis methods for *Cryptosporidium* parasitism and occurrence in hospitalized patients.

Test/Analysis	Number of Samples	Results	Species Identification
CerTest Crypto (rapid chromatographic test)	175	13 positive (7.42%; 95% CI [0.1–68])	*Cryptosporidium* spp.
Modified Ziehl–Neelsen staining (Henriksen and Pohlenz)	175	Confirmation of 13 positives (7.42%)	*Cryptosporidium* spp.
PCR (Nested) and sequencing	4 positive samples *	4 positive (100% of tested samples)	*C. parvum* (2/4; 50%); *C. canis* (2/4; 50%)

* samples positive at CerTest Crypto and Modified Ziehl–Neelsen staining.

**Table 2:** Distribution by age groups of study participants diagnosed with *Cryptosporidium* spp.

Variable		Number of Tested Subjects n = 175	Number of Patients with Positive Test Results n = 13 (%)	OR 95%CI	*p*-Value
**Age group**	<1	32	1 (3.1)	Ref	
	1–5	64	5 (7.8)	2.63 (0.3–23.5)	0.37
	6–10	18	1 (5.6)	1.82 (0.1–31)	0.67
	11–17	4	0 (0)	-	0.72
	18–39	15	1 (6.7)	2.21 (0.1–38)	0.54
	40–59	13	2 (15.4)	5.63 (0.5–68)	0.14
	≥60	29	3 (10.3)	3.58 (0.4–36.5)	0.26

**Table 3:** Occurrence and risk factors of *Cryptosporidium* spp. infection in humans in Romania.

Variables		Number of Tested Subjects n = 175	Number of Patients with Positive Test Results n = 13 (%)	OR 95% CI	*p*-Value
**Area of residence**	Rural	126	6 (4.8)	3.33 (1.1–10.5)	0.03 **
	Urban	49	7 (14.3)		
**Sex**	Female	88	4 (4.6)	2.42 (0.7–8.2)	0.14
	Male	87	9 (10.3)		
**Digestive symptoms**	Yes	94	7 (7.5)	0.99 (0.3–3.1)	0.99
	No	81	6 (7.4)		
Contact with dogs and other animals *	Yes	78	7 (9)	0.67 (0.2–2.1)	0.49
	No	97	6 (6.2)		
**Washing hands after contact with animals**	Yes	135	10 (7.4)	Ref	
	Sometimes	31	2 (6.5)	0.86 (0.2–4.1)	0.85
	No	1	1 (100)	NA	0.0007 **
	No answer	8	0 (0)	NA	0.42
**Drinking tested potable water**	Yes	112	11 (9.8)	Ref	
	No	7	0 (0)	NA	0.39
	Don’t know	56	2 (3.6)	0.34 (0.1–1.6)	0.15

* cats, cows, sheep, goats, pigs, horses, chickens, parrots, ** Statistically significant value, NA—not available

**Table 4:** Multivariate Logistic Regression Results.

Variable	Adjusted OR	95% CI	*p*-Value
**Residence**	1.250	0.353–4.424	0.729
**Sex**	0.462	0.134–1.599	0.223
**Digestive_symptoms**	0.793	0.243–2.580	0.699
**Animal_contact**	1.537	0.408–5.786	0.525
**Handwashing**	0.767	0.133–4.412	0.766
**Potable_water**	0.285	0.058–1.394	0.121

**Table 5:** Characteristics of cases identified with *Cryptosporidium* spp.

No. Crt	Sex	Age (Years)	Rural (R)/Urban (U)	Diagnosis	Diarrhea
**1**	M	36	R	HIV, pulmonary TBC, Enterocolitis with C. difficile	Yes
**2**	M	47	U	Enterocolitis with C. difficile	No *
**3**	F	1	R	Measles	Yes
**4**	M	78	U	Febrile syndrome	No
**5**	F	3	R	Convulsive cough	No
**6**	M	9	U	Febrile syndrome	No
**7**	F	1	U	Febrile syndrome	No
**8**	M	1	U	Rotavirus enterocolitis	Yes
**9**	M	55	U	HIV	No
**10**	M	79	R	Enterocolitis with C. difficile, HTN	Yes
**11**	M	10 months	R	Measles	Yes
**12**	F	2	R	Enterocolitis	Yes
**13**	M	75	U	COVID-19, Parkinson’s disease	No
**Total**	9M + 4F	10 months-78 years old	6R + 7U		6 yes

Legend: * Treated with Vancomycin; HIV—Human Immunodeficiency Virus; HTN—Hypertension; TBC—Tuberculosis.

**Table 6:** Characteristics of the four cases identified with *Cryptosporidium* spp. in Romania.

Characteristics	Case 1	Case 2	Case 3	Case 4
**Infection with:**	*C. canis*	*C. canis*	*C. parvum*	*C. parvum*
**Sex**	Male	Male	Male	Female
**Age**	36	47	75	2
**Area of residence**	Rural	Urban	Urban	Rural
**Date of detection**	2 August 2024	2 December 2024	4 May 2024	4 March 2024
**Type of dwelling**	House	House	House	Apartment
**Animals raising**	Dogs, cats, chickens	Dogs	Dogs	No
**Contact with animals**	Yes	Yes	Yes	No
**Washing hands after contact with animals**	Yes	Yes	Yes	-
**Eating unwashed vegetables and fruits**	No	No	No	No
**Drinking only drinking water**	Yes	Yes	Yes	Yes
**Frequent swimming pools**	No	No	No	Sometimes
**Frequent playgrounds for children**	No	No	No	Yes
**Using public transport**	No	No	Sometimes	Sometimes
**Travels abroad**	No	No	Yes	Sometimes
**Primary diagnosis**	HIV, Pulmonary TBC, Enterocolitis with *Clostridium difficile*	Acute enterocolitis with *Clostridium dificille*	COVID-19 Infection, Parkinson disease	Enterocolitis
**Diarrhea**	Yes	No	No	Yes
**Treatment with antibiotics**	Vancomicin 125 mg + Gentamicin 315 mg + Ceftamil 2 g + Sumetrolim	Vancomicin	No	Augmentin
**Knowledge about *Cryptosporidium***	No	No	No	No

Legend: HIV—Human Immunodeficiency Virus; TBC—Tuberculosis.

## Data Availability

The original contributions presented in this study are included in the article. Further inquiries can be directed to the corresponding authors.

## Supplementary Materials

The following supporting information can be downloaded at: https://www.mdpi.com/article/10.3390/microorganisms13040931/s1, S1: GenBank deposits of the isolates.
