# Supplementary material for: First Detection of Cryptosporidium Canis and Occurrence of Cryptosporidium spp. in Hospitalized Patients in Romania
Source: Microorganisms. 2025 Apr 17;13(4):931. doi: 10.3390/microorganisms13040931 (PMC12029450; doi:10.3390/microorganisms13040931)
Supplement: Supplementary file 1 [file microorganisms-13-00931-s001.zip › microorganisms-3491575-supplementary.pdf]

An official website of the United States government

Here's how you know

Nucleotide

GenBank

Cryptosporidium canis isolate RO_ 560 small subunit ribosomal RNA gene, partial sequence

GenBank: PP979610.1

FASTA Graphics

Go to:

```
LOCUS       PP979610                 792 bp    DNA     linear   INV 08-JUL-2024
DEFINITION  Cryptosporidium canis isolate RO_ 560 small subunit ribosomal RNA
            gene, partial sequence.
ACCESSION   PP979610
VERSION     PP979610.1
KEYWORDS    .
SOURCE      Cryptosporidium canis
  ORGANISM  Cryptosporidium canis
            Eukaryota; Sar; Alveolata; Apicomplexa; Conoidasida; Coccidia;
            Eucoccidiorida; Eimeriorina; Cryptosporidiidae; Cryptosporidium.
REFERENCE   1  (bases 1 to 792)
  AUTHORS   Imre,M., Darabus,R.G., Darabus,G., Darabus,D., Ilie,M. and
            Olariu,R.
  TITLE     First detection of Cryptosporidium canis in human in Romania and
            the identification of risk factors of risk factors
  JOURNAL   Unpublished
REFERENCE   2  (bases 1 to 792)
  AUTHORS   Imre,M., Darabus,R.G., Darabus,G., Darabus,D., Ilie,M. and
            Olariu,R.
  TITLE     Direct Submission
  JOURNAL   Submitted (03-JUL-2024) Parasitology and Parasitic Disease
            Department, University of Life Sciences Timisoara, Timisoara, Calea
            Aradului No. 119, Timisoara, Timis 300645, Romania
COMMENT     ##Assembly-Data-START##
            Sequencing Technology :: Sanger dideoxy sequencing
            ##Assembly-Data-END##
FEATURES             Location/Qualifiers
     source          1..792
                     /organism="Cryptosporidium canis"
                     /mol_type="genomic DNA"
                     /isolate="RO_ 560"
                     /isolation_source="fecal samples"
                     /host="Homo sapiens"
                     /db_xref="taxon:195482"
                     /geo_loc_name="Romania"
     rRNA            <1..>792
                     /product="small subunit ribosomal RNA"
ORIGIN
        1 gtagcgcgct caggagggtc gagctactac ttctggtgcg gcccactcgg atggatgagc
       61 ttggacggta gacgtattgg taccgtctga ctctgaccgg taacggggaa ttagggttcg
      121 attccggaga gggagcctga gaaaggctac cacatctaag gaaggcagca ggcgcgcaaa
      181 ttacccaatc ctaatacagg gaggtagtga caagaaataa caatacagga ctttaacagt
      241 tttgtaattg gaatgagttg agtataaacc cctttacaag tatcaattgg agggcaagtc
      301 tggtgccagc agccgcggta attccagctc caatagcgta tattaaagtt gttgcagtta
      361 aaaagctcgt agttggattt ctgttaataa tttatatata atatttaaca tatttatata
      421 atattaacat aattcatatt actatttata gtatatgaaa ctttactttg agaaaattag
      481 agtgcttaaa gcaggctttt gccttgaata ctagagcatg gaataatatt aaagattttt
      541 atctttctta ttggttctaa gatagaaata atgattaata gggacagttg ggggcatttg
      601 tatttaacag ttagaaggtg aaattcttag atttgttaaa gacaaactaa tgcgaaagca
      661 tttgcccaag gatgttttca ttaatcaaga acgaaagtta ggggatcgaa gacgatcaga
      721 taccgtcgta gtcttaacca taaactatgc caactagaga ttggaggttg ttccttactc
      781 ctcccccctt tg
//
```

An official website of the United States government

Here's how you know

Cryptosporidium canis isolate RO_584 small subunit ribosomal RNA gene, partial sequence

GenBank: PP979613.1


```
LOCUS       PP979613                 578 bp    DNA     linear   INV 08-JUL-2024
DEFINITION  Cryptosporidium canis isolate RO_584 small subunit ribosomal RNA
            gene, partial sequence.
ACCESSION   PP979613
VERSION     PP979613.1
KEYWORDS    .
SOURCE      Cryptosporidium canis
  ORGANISM  Cryptosporidium canis
            Eukaryota; Sar; Alveolata; Apicomplexa; Conoidasida; Coccidia;
            Eucoccidiorida; Eimeriorina; Cryptosporidiidae; Cryptosporidium.
REFERENCE   1  (bases 1 to 578)
  AUTHORS   Imre,M., Darabus,R.G., Darabus,G., Darabus,D., Ilie,M. and
            Olariu,R.
  TITLE     First detection of Cryptosporidium canis in human in Romania and
            the identification of risk factors of risk factors
  JOURNAL   Unpublished
REFERENCE   2  (bases 1 to 578)
  AUTHORS   Imre,M., Darabus,R.G., Darabus,G., Darabus,D., Ilie,M. and
            Olariu,R.
  TITLE     Direct Submission
  JOURNAL   Submitted (03-JUL-2024) Parasitology and Parasitic Disease
            Department, University of Life Sciences Timisoara, Timisoara, Calea
            Aradului No. 119, Timisoara, Timis 300645, Romania
COMMENT     ##Assembly-Data-START##
            Sequencing Technology :: Sanger dideoxy sequencing
            ##Assembly-Data-END##
FEATURES             Location/Qualifiers
     source          1..578
                     /organism="Cryptosporidium canis"
                     /mol_type="genomic DNA"
                     /isolate="RO_584"
                     /isolation_source="fecal samples"
                     /host="Homo sapiens"
                     /db_xref="taxon:195482"
                     /geo_loc_name="Romania"
     rRNA            <1..>578
                     /product="small subunit ribosomal RNA"
ORIGIN
        1 aacggactac cacgagaaag gaaggcagca ggcgcgcaaa ttacccaatc ctaatacagg
       61 gaggtagtga caagaaataa caatacagga ctttaacagt tttgtaattg gaatgagttg
      121 agtataaacc cctttacaag tatcaattgg agggcaagtc tggtgccagc agccgcggta
      181 attccagctc caatagcgta tattaaagtt gttgcagtta aaaagctcgt agttggattt
      241 ctgttaataa tttatatata atatttaaca tatttatata atattaacat aattcatatt
      301 actatttata gtatatgaaa ctttactttg agaaaattag agtgcttaaa gcaggctttt
      361 gccttgaata ctagagcatg gaataatatt aaagattttt atctttctta ttggttctaa
      421 gatagaaata atgattaata gggacagttg ggggcatttg tatttaacag ttagaaggtg
      481 aaattcttag atttggttaa agacaaacta atgcgaaagc attgccaagg gatgttttca
      541 ttaatcaaga acgaaagtta ggggatcgaa gacgatca
//
```

An official website of the United States government
Here's how you know

Nucleotide

GenBank

Cryptosporidium parvum isolate RO_582 small subunit ribosomal RNA gene, partial sequence

GenBank: PP979614.1

FASTA Graphics

Go to:

```
LOCUS       PP979614                 760 bp    DNA     linear   INV 08-JUL-2024
DEFINITION  Cryptosporidium parvum isolate RO_582 small subunit ribosomal RNA
            gene, partial sequence.
ACCESSION   PP979614
VERSION     PP979614.1
KEYWORDS    .
SOURCE      Cryptosporidium parvum
  ORGANISM  Cryptosporidium parvum
            Eukaryota; Sar; Alveolata; Apicomplexa; Conoidasida; Coccidia;
            Eucoccidiorida; Eimeriorina; Cryptosporidiidae; Cryptosporidium.
REFERENCE   1  (bases 1 to 760)
  AUTHORS   Imre,M., Darabus,R.G., Darabus,G., Darabus,D., Ilie,M. and
            Olariu,R.
  TITLE     First detection of cryptosporidium canis in human in Romania and
            the identification of risk factors of risk factors
  JOURNAL   Unpublished
REFERENCE   2  (bases 1 to 760)
  AUTHORS   Imre,M., Darabus,R.G., Darabus,G., Darabus,D., Ilie,M. and
            Olariu,R.
  TITLE     Direct Submission
  JOURNAL   Submitted (03-JUL-2024) Parasitology and Parasitic Disease
            Department, University of Life Sciences Timisoara, Timisoara, Calea
            Aradului No. 119, Timisoara, Timis 300645, Romania
COMMENT     ##Assembly-Data-START##
            Sequencing Technology :: Sanger dideoxy sequencing
            ##Assembly-Data-END##
FEATURES             Location/Qualifiers
     source          1..760
                     /organism="Cryptosporidium parvum"
                     /mol_type="genomic DNA"
                     /isolate="RO_582"
                     /isolation_source="fecal samples"
                     /host="Homo sapiens"
                     /db_xref="taxon:5807"
                     /geo_loc_name="Romania"
     rRNA            <1..>760
                     /product="small subunit ribosomal RNA"
ORIGIN
        1 atcattcaag tttctgacct atcagcttta gacggtaggg tattggccta ccgtggcaat
       61 gacgggtaac ggggaattag ggttcgattc cggagaggga gcctgagaaa cggctaccac
      121 atctaaggaa ggcagcaggc gcgcaaatta cccaatccta atacagggag gtagtgacaa
      181 gaaataacaa tacaggactt tttggttttg taattggaat gagttaagta taaacccctt
      241 tacaagtatc aattggaggg caagtctggt gccagcagcc gcggtaattc cagctccaat
      301 agcgtatatt aaagttgttg cagttaaaaa gctcgtagtt ggatttctgt taataattta
      361 tataaaatat tttgatgaat atttatataa tattaacata attcatatta ctatatattt
      421 tagtatatga aattttactt tgagaaaatt agagtgctta aagcaggcat atgccttgaa
      481 tactccagca tggaataata ttaaagattt ttatctttct tattggttct aagataagaa
      541 taatgattaa tagggacagt tgggggcatt tgtatttaac agtcagaggt gaaattctta
      601 gatttgttaa agacaaacta atgcgaaagc atttgccaag gatgttttca ttaatcaaga
      661 acgaaagtta ggggatcgaa gacgatcaga taccgtcgta gtcttaacca taaactatgc
      721 caactagaga ttgggaggtt gttcctactc tcccccccttt
//
```

An official website of the United States government
Here's how you know

Nucleotide

Cryptosporidium parvum isolate RO_583 small subunit ribosomal RNA gene, partial sequence

GenBank: PQ047137.1

FASTA Graphics

Go to:

```
LOCUS       PQ047137                 813 bp    DNA     linear   INV 23-JUL-2024
DEFINITION  Cryptosporidium parvum isolate RO_583 small subunit ribosomal RNA
            gene, partial sequence.
ACCESSION   PQ047137
VERSION     PQ047137.1
KEYWORDS    .
SOURCE      Cryptosporidium parvum
  ORGANISM  Cryptosporidium parvum
            Eukaryota; Sar; Alveolata; Apicomplexa; Conoidasida; Coccidia;
            Eucoccidiorida; Eimeriorina; Cryptosporidiidae; Cryptosporidium.
REFERENCE   1  (bases 1 to 813)
  AUTHORS   Imre,M., Darabus,R.G., Darabus,G., Darabus,D., Ilie,M. and
            Olariu,R.
  TITLE     First detection of cryptosporidium canis in human in Romania and
            the identification of risk factors of risk factors
  JOURNAL   Unpublished
REFERENCE   2  (bases 1 to 813)
  AUTHORS   Imre,M., Darabus,R.G., Darabus,G., Darabus,D., Ilie,M. and
            Olariu,R.
  TITLE     Direct Submission
  JOURNAL   Submitted (18-JUL-2024) Parasitology and Parasitic Disease
            Department, University of Life Sciences Timisoara, Timisoara, Calea
            Aradului No. 119, Timisoara, Timis 300645, Romania
COMMENT     ##Assembly-Data-START##
            Sequencing Technology :: Sanger dideoxy sequencing
            ##Assembly-Data-END##
FEATURES             Location/Qualifiers
     source          1..813
                     /organism="Cryptosporidium parvum"
                     /mol_type="genomic DNA"
                     /isolate="RO_583"
                     /isolation_source="fecal samples"
                     /host="Homo sapiens"
                     /db_xref="taxon:5807"
                     /geo_loc_name="Romania"
     rRNA            <1..>813
                     /product="small subunit ribosomal RNA"
ORIGIN
        1 gaagggggtt gtgtattatt agataaagaa ccaatataat tggtgactca taataacttt
       61 acggatcaca ttaaatgtga catatcattc aagtttctga cctatcagct ttagacggta
      121 gggtattggc ctaccgtggc aatgacgggt aacggggaat tagggttcga ttccggagag
      181 ggagcctgag aaacggctac cacatctaag gaaggcagca ggcgcgcaaa ttacccaatc
      241 ctaatacagg gaggtagtga caagaaataa caatacagga ctttttggtt ttgtaattgg
      301 aatgagttaa gtataaaccc ctttacaagt atcaattgga gggcaagtct ggtgccagca
      361 gccgcggtaa ttccagctcc aatagcgtat attaaagttg ttgcagttaa aaagctcgta
      421 gttggatttc tgttaataat ttatataaaa tattttgatg aatatttata taatattaac
      481 ataattcata ttactatata ttttagtata tgaaatttta ctttgagaaa attagagtgc
      541 ttaaagcagg catatgcctt gaatactcca gcatggaata atattaaaga tttttatctt
      601 tcttattggt tctaagataa gaataatgat taatagggac agttgggggc atttgtattt
      661 aacagtcaga ggtgaaattc ttagatttgt taaagacaaa ctaatgcgaa agcatttgcc
      721 aaggatgttt tcattaatca agaacgaaag ttaggggatc gaagacgatc agataccgtc
      781 gtagtcttaa ccataaacta tgccaactag aga
//
```
